# A new approach to deposit homogeneous samples of asbestos fibres for toxicological tests *in vitro*

**DOI:** 10.3389/fchem.2023.1116463

**Published:** 2023-02-14

**Authors:** Giancarlo Della Ventura, Ahmad Rabiee, Augusto Marcelli, Salvatore Macis, Annalisa D’Arco, Gianluca Iezzi, Francesco Radica, Federico Lucci

**Affiliations:** ^1^ Department of Science, Roma Tre University, Rome, Italy; ^2^ INFN, National Institute of Nuclear Physics, Frascati, Italy; ^3^ INGV, National Institute of Geophysics and Volcanology, Rome, Italy; ^4^ RICMASS, Rome International Center for Materials Science Superstripes, Rome, Italy; ^5^ Department of Physics, Sapienza University of Rome, Rome, Italy; ^6^ Department of Engineering and Geology, University of Chieti, Rome, Italy; ^7^ Department of Earth and Geoenvironmental Sciences, University of Bari, Bari, Italy

**Keywords:** asbestos fibres, deposition, toxicological experiments, microdrop method, optical and SEM images, image processing

## Abstract

In this paper we describe the results obtained with a novel method to prepare depositions of asbestos fibres for toxicological tests *in vitro*. The technique is based on a micro-dispenser, working as an inkjet printer, able to deposit micro-sized droplets from a suspension of fibres in a liquid medium; we used here a highly evaporating liquid (ethanol) to reduce the experimental time, however other solvents could be used. Both the amount and spatial distribution of fibres on the substrate can be controlled by adjusting the parameters of the micro-dispenser such as deposition area, deposition time, uniformity and volume of the deposited liquid. Statistical analysis of images obtained by optical and scanning electron microscopy shows that this technique produces an extremely homogeneous distribution of fibers. Specifically, the number of deposited single fibres is maximized (up to 20 times), a feature that is essential when performing viability tests where agglomerated or untangled fibrous particles need to be avoided.

## 1 Introduction

Asbestiform fibres are inhalable minerals with length from few up to tens of µm and with lateral dimension generally <1 μm, characterized by an aspect ratio defined as the length/width ratio >3 (e.g., Belluso et al., 2017; Gualtieri, 2017; Vigliaturo et al., 2021). Six natural crystalline silicate minerals are regulated as asbestos; the list includes the serpentine-group mineral chrysotile and five amphiboles (see Gunter et al., 2007; Ballirano et al., 2017): tremolite asbestos, actinolite asbestos, anthophyllite asbestos, grunerite asbestos (amosite) and crocidolite. They are common minerals in a wide typology of igneous and metamorphic rocks, as well as in soils derived from rocks disaggregation (e.g., Iezzi et al., 2007, Vignaroli et al., 2014; Gunter, 2018; Lucci et al., 2018; Di Giuseppe et al., 2012; Petriglieri et al., 2021). In the last few decades other fibrous minerals as well as elongated man-made materials have been recognized to induce adverse health effects (see the review by Turci et al., 2017).

Due to their aerodynamic diameter, when airborne these particles may be inhaled, translocated across upper airways, deposited in the lower respiratory system and in the time interact with pleural and lung tissues and cells, ultimately leading to lung cancer and mesothelioma (e.g., Skinner et al., 1988; Hessel et al., 2005; Capella et al., 2017; Turci et al., 2017). Many studies (e.g., Sturm and Hofmann, 2009; Hofmann, 2011) on asbestos pointed that differences in parameters like concentration, size (lengths vs. widths), aspect ratio (length/width), spatial distribution, agglomeration (impingement or aggregation) of fibres and mineralogy or crystal-chemistry (chemical composition and molecular structure), or even their grinding (Salamatipour et al., 2016; Turci et al., 2017) may induce variable health effects in deep lung apparatus (Yao et al., 2017 and reference therein). Typically, short fibres (<5–10 µm) may be phagocytized, whereas fibres longer than 10 µm are not easily engulfed by macrophages (Fubini and Fenoglio, 2007; Carbone et al., 2013; Yao et al., 2013; Oury et al., 2014; Bursi Gandolfi et al., 2016); this aspect is considered as the cause for cell deaths, chronic inflammation, release of reactive oxygen species (ROS) and formation of asbestos bodies (Aust et al., 2011; Bursi Gandolfi et al., 2016). The crystal-chemistry of fibres affects their toxicity, controlling dissolution behaviors, surface electric charge transfer and redox reactions with the lung cells (Vigliaturo et al., 2022) and may also play a role on the final texture, morphology, fragmentation and splitting (Vigliaturo et al., 2021) of the mineral.

Generally *in vitro* tests are performed by depositing fibres dispersed in a liquid medium, usually a cell culture medium, on a substrate which is placed in well plates where cells are grown. Their toxicity is addressed *via* different methods (e.g., Duncan et al., 2014; Pietrofesa et al., 2016; Yao et al., 2017) typically based on cell mortality (viability). However, understanding how asbestos interact with living cells requires control of the textural features of the used fibres to obtain reproducible results (Chipera et al., 1993; Yao et al., 2017). To partially reduce the incertitude on this issue, several methods have been proposed based on filtering, sieving or magnetic separation of the powdered material used to prepare the suspensions (e.g., Vigliaturo et al., 2020 and references therein). The morphological distribution of particles/fibers and their depositions is addressed *via* image analysis through relatively user-friendly software(s) (Yao et al., 2017; Merico et al., 2020; Vigliaturo et al., 2020).

The most intriguing result of the work of Yao et al. (2017) is that the distribution of particles in the deposition is extremely different for increasing amounts in the suspension: at very low concentration (mg/L < 1) fibres are well separated, whereas for mg/L > 10 an increasing impingement and formation of large bundles is observed. The development of agglomeration textures was found to have direct impact on the toxicity of the system, with the surprising conclusion that the increasing rate of fibres loads induces only a limited increasing of *in vitro* toxicity. This feature could be explained simply based on the fact that lung cells had more difficulty to interact with agglomerated fibres. An additional important result of the work of Yao et al. (2017) was also that textures of asbestos may have an effect also on the chemical potential of their surface. Phases with the same composition, length and width can change drastically their surface activity as a function of their proportion of single *versus* agglomerated fibres.

The hypothesis that toxic effects *in vitro* are mainly imposed by the number of single fibres in the starting cell culture substrate prompted the present research aimed at developing a new deposition method based on a micro-drop dispenser.

**FIGURE 1:**
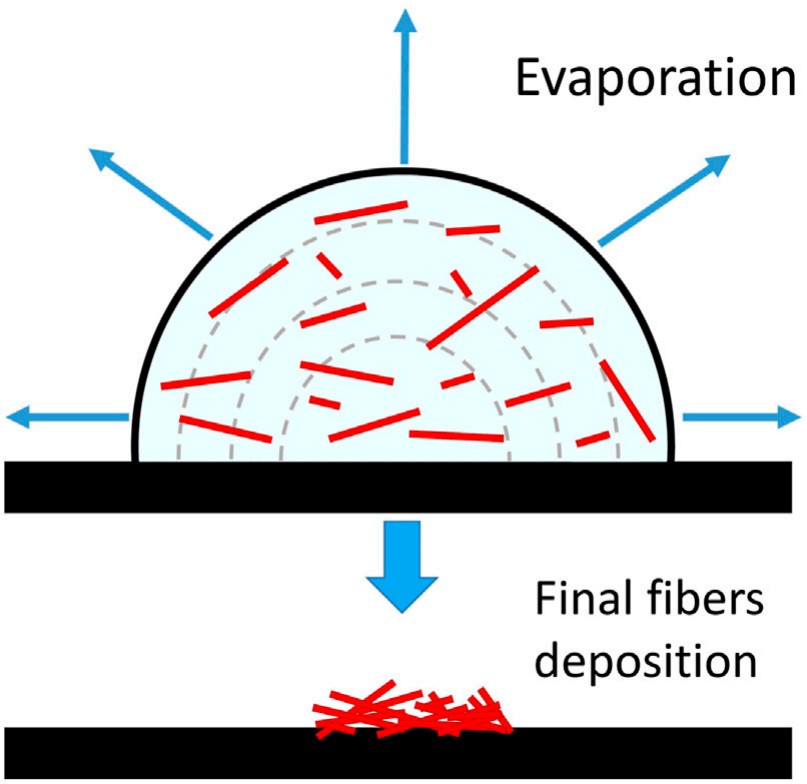
The deposition process of small particles from an evaporating solution on a hydrophobic surface with a negligible drop-surface interaction (redrawn from Macis et al., 2018).

The main drawbacks of the evaporation method typically used to prepare samples for cell-culture tests are (1) the inhomogeneity of the deposition and (2) the usually long evaporation time of the suspension. Feature (2) depends mainly by droplet size and type of solvent, while feature (1) depends on the evaporation dynamics of the drop on the surface where it is deposed. Assuming a negligible drop-surface interaction, as for water-based liquids on a hydrophobic substrate, during the evaporation the contact angle of the edge of the drop remains constant (see Macis et al., 2018), the shape remains spherical but the contact area between liquid and surface continuously decreases. In such a case, due to the stain effect, the deposition of fibres generates bundles and aggregates within a small area, particularly at high concentrations (Figure 1).

In this study, we prepared two depositions starting from the same suspension, by using either a conventional micropipette or a dispenser able to deposit micro-sized droplets, a technology similar to that used by inkjet printers. We demonstrate the efficiency of this latter method by comparing the distribution of fibers/particles on the substrate *via* analysis of images collected by optical and SEM microscopies.

## 2 Materials and methods

### 2.1 The used fibres

**FIGURE 2:**
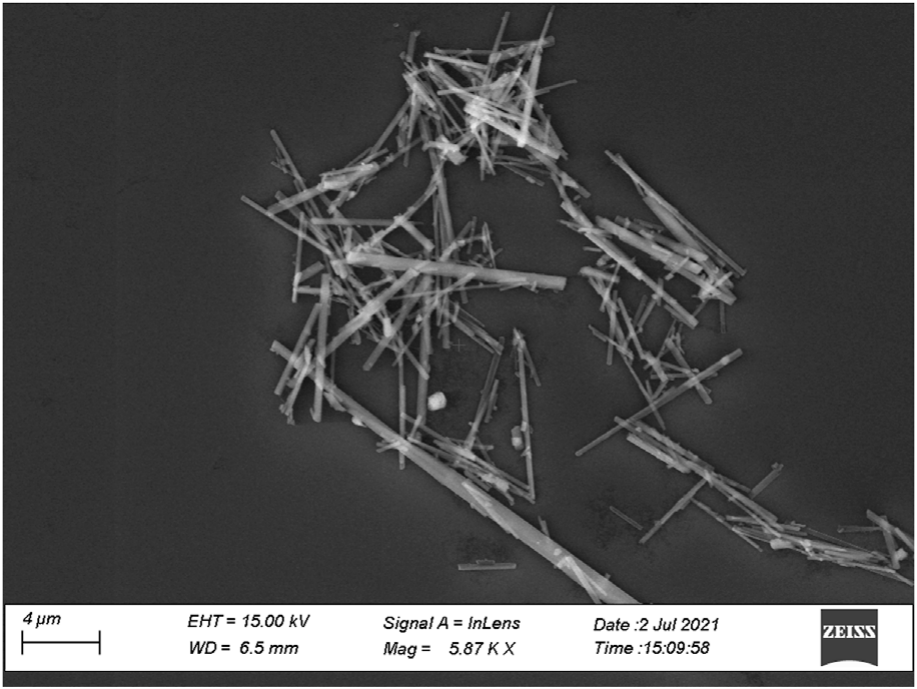
Secondary electrons SEM microphotograph of the synthetic amphibole used for the present study.

For our experiments we used a synthetic amphibole powder prepared by Della Ventura et al. (1997). This compound, Ni-richterite [Na(NaCa)Ni_5_Si_8_O_22_(OH)_2_], does not exist in nature therefore it is not included in the regulated asbestos list. It has been chosen for the tests because it was available as a powder consisting of almost mono-dimensional, very thin and needle-like crystals (Figure 2) with length ranging between 5 and 15 µm and width <1 μm, thus corresponding dimensionally and morphologically to asbestos amphiboles (e.g., Belluso et al., 2017; Vigliaturo et al., 2021). One point to be stress here is that the terminology used in asbestos studies is often confusing because several disciplines (geology, mineralogy, industry, medicine, regulatory agencies etc.) have developed their own nomenclature somewhat independently. Accordingly, the term “asbestiform” is commonly used to describe minerals having longitudinal parting that can be split into individual fibres; the term “fibre” is used for materials that, besides having an elongated morphology, can be bent under force, while the terms “particle” and “fragment” are used for small amphibole crystals matching the above textural criteria (Gunter et al., 2007). In our work, we used a material that cannot be defines as “asbestos” being synthetic and chemically different from the regulated species, however it has all morphological and physical properties of asbestos, allowing it to be termed “asbestiform”.

### 2.2 Dispersion of fibres on the substrate

Five milligrams of powder were suspended in 50 ml of Merck^®^ pure ethanol. Note that this solvent is not suitable for actual experiments with cells because it may induce cytotoxic effects; it was used here because of its fast evaporation time (evaporation enthalpy 42.4 kJ/mol at 303 K: Van Ness et al., 1967) that guarantees quick and efficient depositions for further image analysis.

**FIGURE 3:**
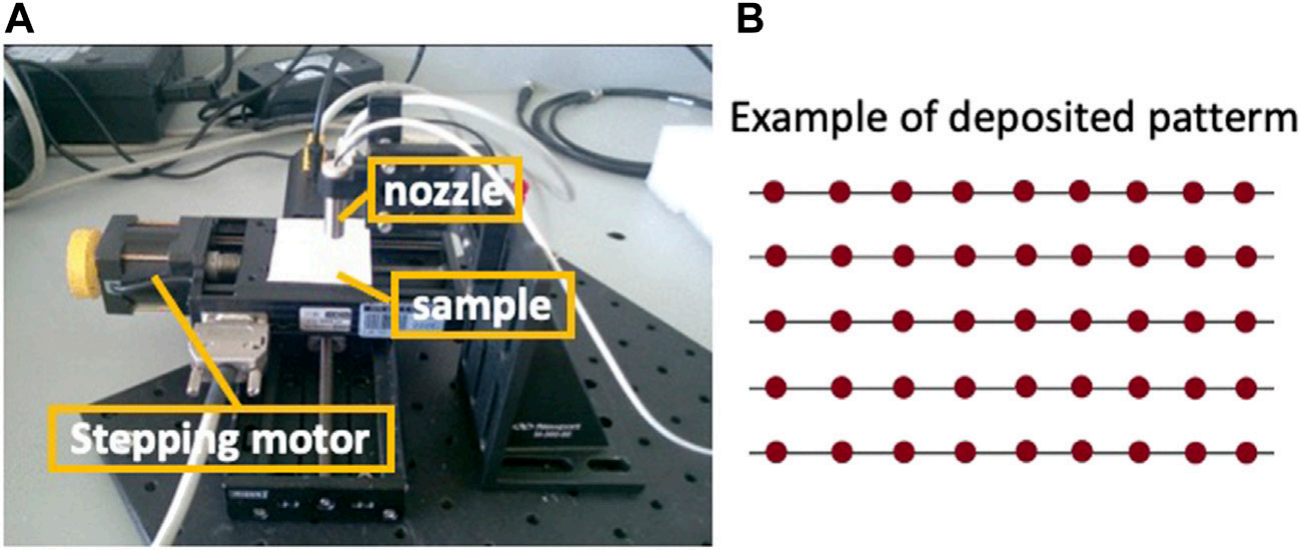
The microdrop device **(A)** and a schematic example of droplets (red spots) deposition pattern **(B)**, where the X-Y distances can be adjusted at any values *via* the used software (see text for details).

The prepared suspension was transferred on silicon substrates, by using both the conventional pipette (hereafter “drop” method) and the “micro-drop” device. Silicon substrates were chosen because they proved to be efficient materials for cell-fibres interaction tests (Yao et al., 2017). They could be directly used in this study for both optical and SEM imagery without any further preparation. For the micro-drop depositions, we used the MD-K-130 instrument of Microdrop Technologies (GmbH Norderstedt, Germany, Figure 3), equipped with an inner nozzle diameter of 70 µm that generates single droplets with a volume of 180 pL (1% volume repeatability), with a variable drop rate from 1 to 2000 Hz. This device, designed for advanced micro-dispensing, inkjet printing and R&D applications (see https://www.microdrop.de) allows to control with high precision the pattern of droplets deposited on the substrate at well-defined and repeatable positions (see layout in Figure 3, right). The method can ensure also sterile working conditions once the metallic nozzle is efficiently cleaned and kept. During the deposition, the dispensing head remains fixed, while the substrate moves along X-Y trajectories thanks to two translation slides assembled perpendicularly, allowing the positioning control of the substrate with an accuracy of ±1 µm. The whole system is handled by a code written in LabView^®^ (a graphical programming environment), permitting the control of several parameters such as deposition area, deposition time, uniformity and amount of deposited liquid. Therefore, the fibres/liquid ratio to be used for cell-culture experiments depends on the parameters selected for the deposition. Just like an inkjet printer, we can start from a single deposition pattern where we release the minimum dose and may increase the amount of fibres with additional depositions on the same (or different) locations. In other words, incremental amounts of fibres on the substrates for different viability tests can be prepared by multiple depositions from the same suspension instead of preparing different suspensions.

### 2.3 Image analysis of the substrates

The depositions were investigated by reflected-light optical microscopy and scanning electron microscopy (SEM): optical images were acquired by using a Leica optical system Z16 APO, while SEM images were acquired with a Zeiss FE-SEM Gemini III instrument operated at 15 kV accelerating voltage and 40 nA beam current.

Image processing and analysis were performed by using the LabView^®^ vision assistant software. Selected images were first converted to grayscale, applying both convolution and smoothing filters, then binarized by applying an adequate thresholding range. The converted files were analysed through the “Particle Analysis” tool build in the LabView^®^ environment to count particles in each generated binary image and to obtain the morphological features for each object. In our analysis, all extremely small particles (<2 µm in length) were included into a single class, because they could not be efficiently discriminated from the background. However, this simplification does not affect our conclusions regarding the suitability of the depositions for toxicological tests, because all dimensional classes with lengths <5 µm are not considered to be carcinogenic (Stanton et al., 1981; Davis and Jones, 1988), although the pathogenic potential of short fibres cannot be totally excluded (Pollard, 2020). They are included in the following plots for statistical purposes to describe the distributions obtained with the two methods, i.e., drop vs. microdrop.

Asbestos counting on both optical (collected with ×40 objective) and SEM images was performed manually following the NIOSH fibre counting Method 7400 (Ashley and O'Connor, 2017) that recommends, based on the Asbestos International Association (AIA) guidelines, that objects with these specific features are fibres: (i) longer than 5 µm along their elongation axes, (ii) width <3 μm, (iii) length/width ratio >3, (iv) have no sticking particle larger than 3 µm in diameter; in addition, (v) crossed fibres are counted individually, (vi) branched fibres are counted as a single fibre, (vii) entangled fibres are not counted.

## 3 Results and discussion

**FIGURE 4:**
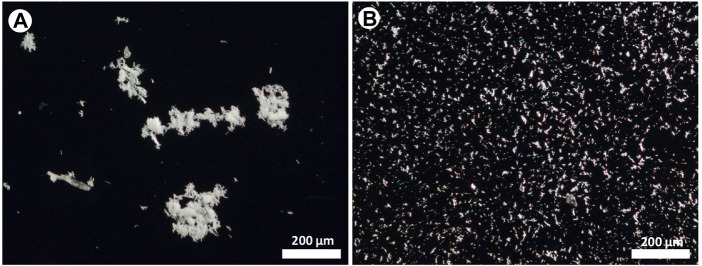
Optical images of the asbestos depositions obtained with **(A)** the pipette (drop) and **(B)** the micro-drop methods, respectively. Optical images taken with ×10 objective lens.

Figure 4 compares images taken after the deposition of fibres from the same solution by using the conventional pipette (drop) method and the micro-drop technique. These images are taken at low magnification (×10 objective) to cover a large field of view for a better and general visual comparison. In the first case (Figure 4A), an extremely heterogeneous distribution of fibres, mainly consisting of large agglomerates, entangled bundles hundreds of µm in size is observed, while the sequential shot of micro-drops yielded a highly homogeneous distribution (Figure 4B) with small objects few tenths of µm in size and single fibres.

### 3.1 Analysis of the depositions *via* image processing

**FIGURE 5:**
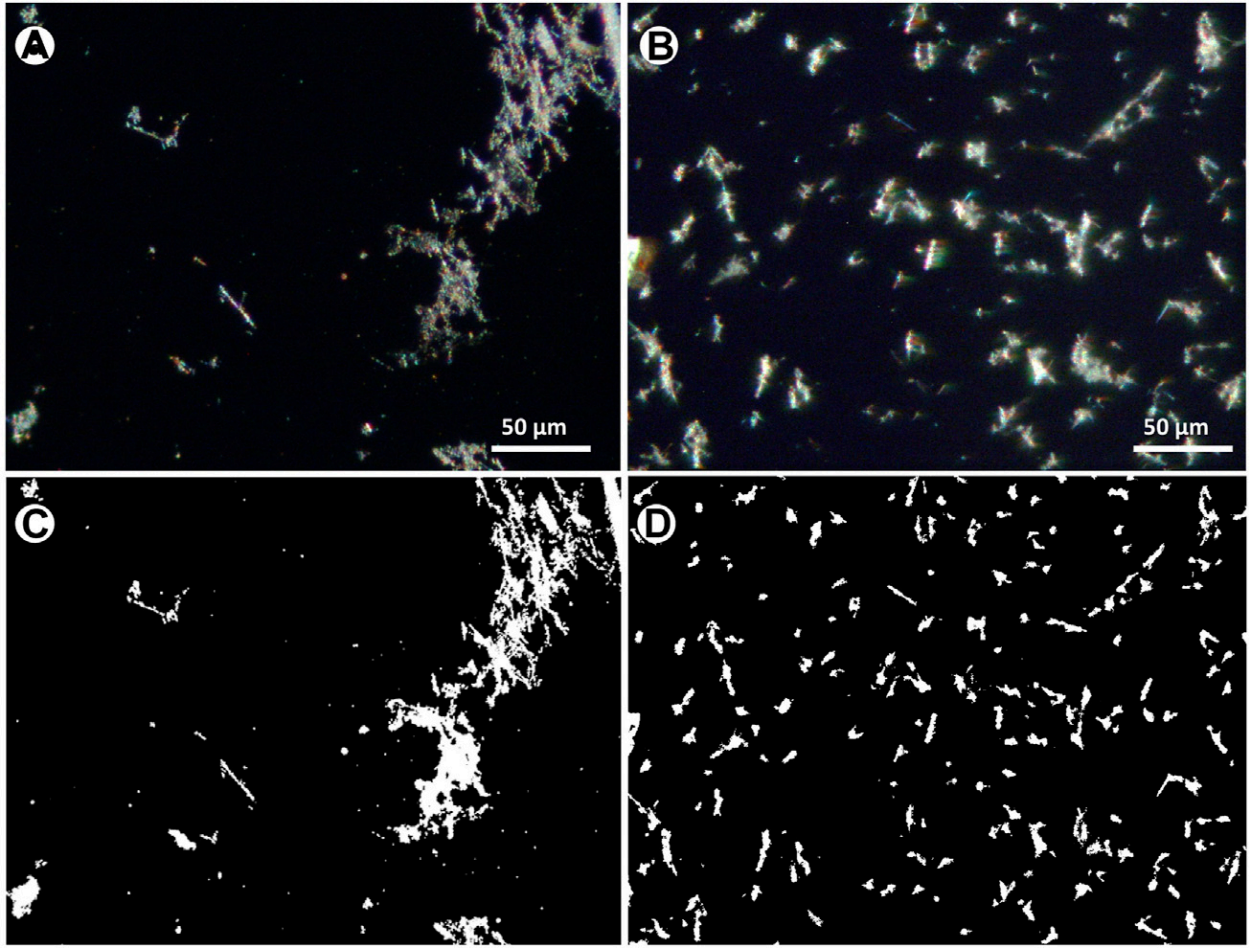
Optical images from the same sample of Figure 4, at ×40 magnification, showing the distributions of **(A)** drop and **(B)** microdrop methods **(C**, **D)** corresponding binary images after thresholding and binarization.

The distribution of fibres resulting from the above-described methods was investigated through the analysis of optical images (Figure 5). The analysis aimed at quantifying the textural parameters such as homogeneity of the deposition, amount and types of entangling fibres, preferential orientation of the particles, number of single fibres etc. An example of phase segmentation and binarization of the images obtained by the drop and the micro-drop depositions are given in Figure 5. Full numerical data provided by the analytical routine are given in the Supplementary Table S1 while a summary of the number of counted objects and their total area with respect to the images, is given in Table 1.

**TABLE 1:** Results of the image processing analysis for representative images collected on the samples obtained using the drop and the micro-drop deposition methods, respectively. Full results are given in (Supplementary Table S1).

Method	Drop method		Microdrop method	
Row	Objects counts	Coverage (%)	Objects counts	Coverage (%)
image 1	7	2.9	192	5.4
image 2	5	10.4	80	5.7
image 3	5	5.9	122	4.7
image 4	20	3.4	206	6.9
image 5	36	1.9	240	6.6
image 6	37	8.1	242	5.5
image 7	19	4.6	291	5.9
image 8	31	3.6	172	5.6
image 9	29	6.9	352	6.8
image 10	32	1.7	165	2.6
image 11	36	3.2	249	6.1
image 12	32	3.9	196	3.3
image 13	29	1.1	212	1.9
image 14			245	2.6
Sum	317		2964	
mean	24	4.4	212	5.0
RSD (%)	48%	59	31%	32
in 1 mm^2^	345		2991	

The counting obtained by using the drop method shows an average surface coverage (Table 1) of 4.4% (1.1–10.4%) with a mean of 24 objects per image (5–37), while the sample deposited using the micro-drop method shows an average coverage of 5.0% (1.9–6.8%) with a mean of 212 objects per image (80–352) (Table 1). Therefore, although a very similar amount of material is deposed in the same area (4.4% vs*.* 5%), the micro-drop method yielded a much better distribution, clearly recognized in Figure 5 with a far larger (9 times higher) number of counted objects.

**FIGURE 6:**
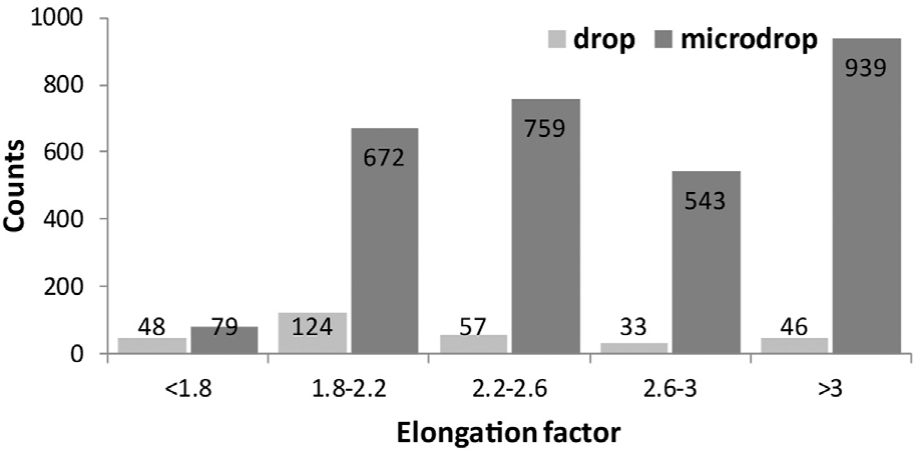
Histograms showing the elongation factor (EF, length/width) of the identified objects for both drop and microdrop deposition methods, respectively. The class of objects with EF > 3 contains fibres according to AIA guidelines. Counts are normalised to mm^2^.

The elongation factor (EF), expressed as their length/diameter ratio is displayed in Figure 6 against the normalized number (to 1 mm^2^) of objects; 46 *versus* 930 particles with EF > 3 (a class including asbestiform particles) are counted for the depositions obtained with the drop and micro-drop methods, respectively. In more detail, for the classical drop method the fibrous particles are about 15% out of the total counted particles (169 over 1144), whereas for the micro drop they are 31% (930 over 2964) (Table 2). This large difference straightforwardly indicates that the depositions obtained *via* the conventional micropipette method may produce unrealistic results in toxicology test. Moreover, we note also that with the drop deposition ∼60% of the total objects (Figure 6; Table 2) have a very low elongation factor (EF < 2.2), meaning that the deposited particles are extremely entangled, i.e., forming very stubby objects. However, it must be underlined here that the category “objects with elongation factor >3” in Figure 6 includes, besides objects <3 µm in length also objects with a diameter >3 μm, that cannot be considered asbestos according to the AIA rules cited above.

**TABLE 2:** Results of the image processing analysis for samples obtained using the drop and the micro-drop deposition methods, respectively. Full results are given in (Supplementary Table S1).

Method	Drop				Microdrop			
	*Class*	*Frequency (counts)*	*Frequency* (*normalised to 1 mm* ^*2*^)	*Cumulative* (%)	*Class*	*Frequency (counts)*	*Frequency* (*normalised to 1 mm* ^*2*^)	*Cumulative* (%)
Elongation factor	<1.8	179	48	15.6	<1.8	78	79	2.63
	1.8–2.2	460	124	55.9	1.8–2.2	666	672	25.10
	2.2–2.6	212	57	74.4	2.2–2.6	752	759	50.47
	2.6–3	124	33	85.2	2.6–3	538	543	68.62
	>3	169	46	100.0	>3	930	939	100.00
Area (µm^2^)	<2	286	77	25.0	<2	1545	1559	52.13
	2–5	221	60	44.3	2–5	385	389	65.11
	5–10	154	42	57.8	5–10	266	268	74.09
	10–15	82	22	64.9	10–15	132	133	78.54
	15–20	57	15	69.9	15–20	107	108	82.15
	>20	344	93	100.0	>20	529	534	100.00
Length (µm)	0–2	273	74	23.9	0–2	1342	1354	45.28
	2–3	192	52	40.6	2–3	320	323	56.07
	3–4	123	33	51.4	3–4	209	211	63.12
	4–5	85	23	58.8	4–5	168	170	68.79
	>5	471	127	100.0	>5	925	933	100.00

**FIGURE 7:**
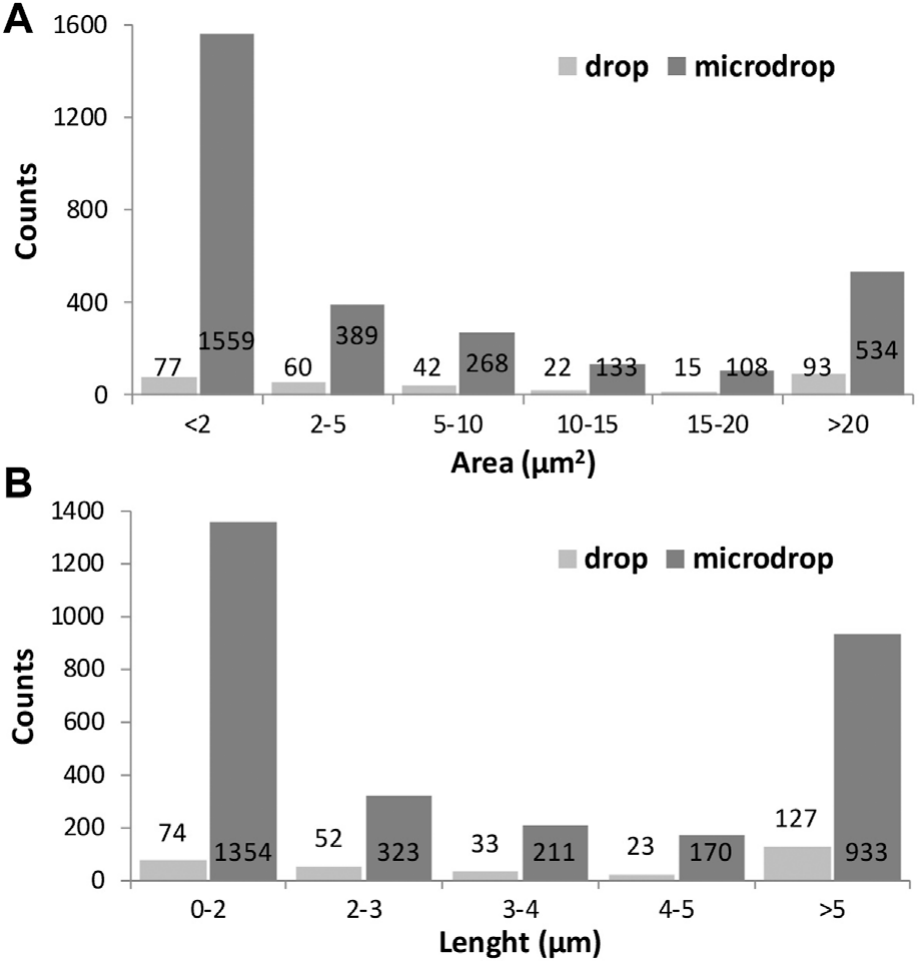
Histograms showing the size distribution of the identified objects represented by their area in µm^2^ **(A)** and their maximum length in µm **(B)** for drop and microdrop depositions, respectively. Counts are normalised to mm^2^.

The size distribution of objects identified through the image analysis is displayed in Figure 7, in terms of measured area in µm^2^ (Figure 7A) or length (7b) for drop and micro-drop depositions, respectively. From the data in Table 2 and the images in Figure 7 it is evident that the number of small objects (i.e., those having an area <2 μm^2^) deposited by the drop method is ∼20 times lower than the number of similar objects deposited by the micro-drop method (77 vs. 1559, corresponding to 25% and 52% out of the total counts, respectively). However, the parameter “area” derived *via* the image processing and plotted in Figure 7 does not consider the shape of the objects. In the present case, since the used powder contains (see Figure 1) only very fibrous crystallites, any “small object” can be considered as a single asbestos fibre. The same kind of information is obtained by inspection of Figure 7B where the number of counts is plotted against their maximum length, obtained as the longest diameter of the rectangle contouring the object. Moreover, the number of objects with length <5 µm is more than seven times lower with the drop than with the micro-drop method, respectively (127 vs. 933, Table 2).

### 3.2 Fibre counting

A very common source of error in the analysis of images is related to the classic workflow based on background subtraction and color thresholding (segmentation), particularly when objects to be identified share colors and hue comparable to the background. This problem is exacerbated when treating optical images due to their intrinsic relatively low resolution.

**FIGURE 8:**
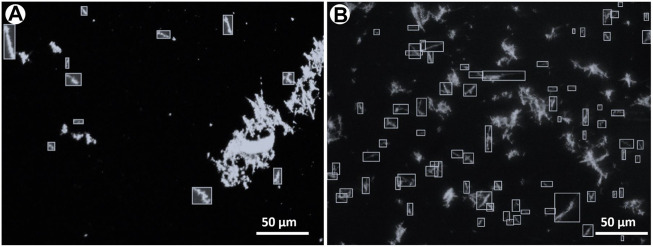
The same optical microscopy images as in Figure 5 where asbestiform fibres have been manually annotated as bounding boxes for counting. The choice of the fibres to annotate has been based on NIOSH (Ashley and O'Connor, 2017) counting rules, see text for explanation. **(A)** Drop and **(B)** micro-drop depositions.

**FIGURE 9:**
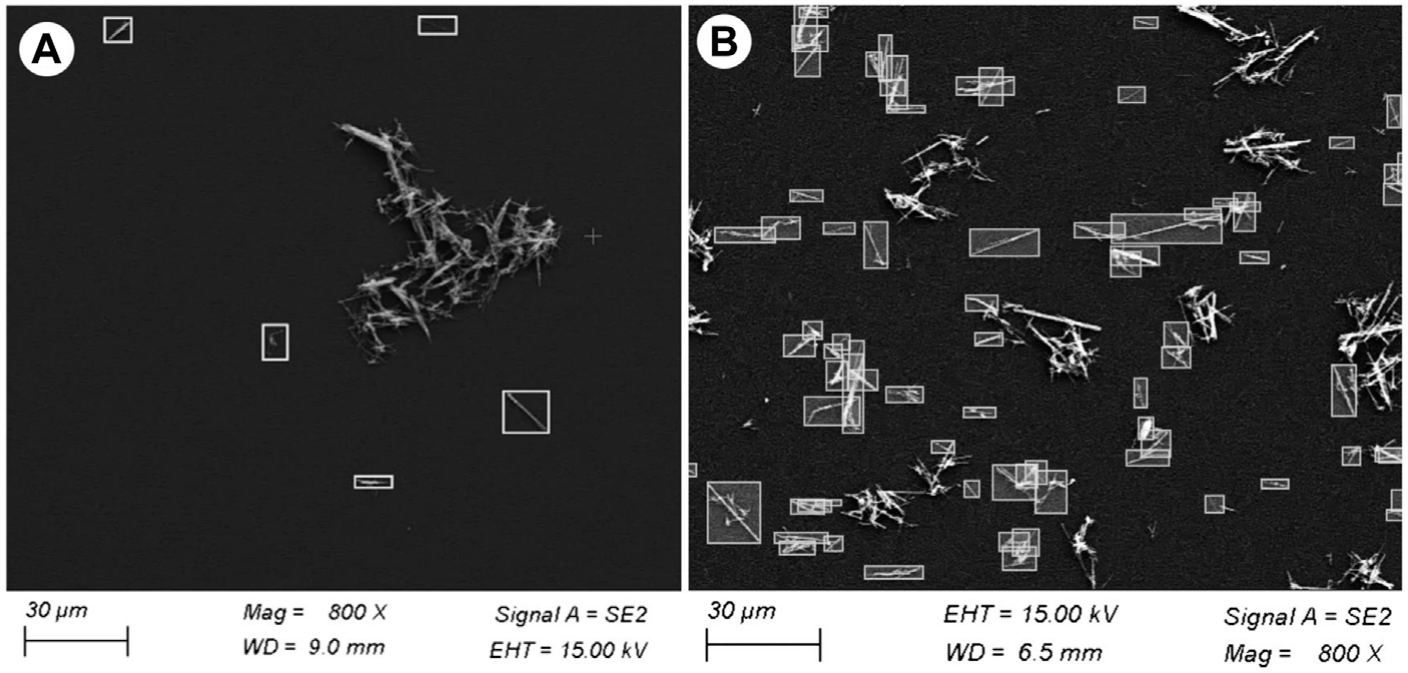
Selected SEM images showing asbestiform fibres manually annotated as bounding boxes for counting. The choice of the fibres to annotate has been based on NIOSH (Ashley and O'Connor, 2017) counting rules, see text for explanation. **(A)** Drop and **(B)** micro-drop depositions. Note that although the magnification of both images is the same (×800), in the micro-drop deposition the distribution of the particles is extremely more diffused.

In environmental monitoring, the exposure risk to airborne asbestos is assessed by counting the fibres contained in a defined volume of sampled air; the technique commonly used for this task is the manual counting on phase contrast microscopy (PCM), particularly in indoor applications, or in SEM images. The important point here is that the asbestos counting needs to follow precise and standardized rules, such as the protocol recommended by the NIOSH Method 7400 (Ashley and O'Connor, 2017; see also https://www.cdc.gov/niosh/nmam/pdf/7400.pdf). Such possibility is not provided by standard image processing packages and for this reason asbestos counting for environmental purposes is usually done manually. To complement the statistical data discussed above, we used the same procedure on our depositions with the goal of verifying how many single particles on the substrates can be morphologically considered as asbestos fibres according to the NIOSH recommendations. This information is essential in providing robust additional evidence for the suitability of the obtained depositions for foreseen real toxicological tests. In the annotated examples (Figures 8, 9), the number and fraction of single asbestiform fibres in the micro-drop deposition is clearly much higher than in the drop deposition. Normalizing to the total studied areas, the counted fibres in the optical images are 92 vs. 1557 fibres/mm^2^ for drop and micro-drop samples (∼18 times higher), respectively (Table 3). The same calculations for SEM images (Table 3) yielded a similar result: 65 vs. 1916 fibres/mm^2^ (∼29 times higher). An intriguing point from Table 3 is that, for the drop deposition, the number of single fibres for mm^2^ for the optical images is slightly higher in comparison to the SEM images, although reasonably similar (92 vs. 65, Table 3). This is explained by considering the larger inspected area (3.68 mm^2^ for optical vs. 2.08 mm^2^ for SEM, Table 3) for a sample that is extremely heterogeneous (Figure 8), hence the counting is underestimated because of the smaller area imaged by SEM. The numbers of Table 3 could also be affected by an error introduced by the operator that, during the manual counting, needs to avoid the entangled particles. This being the case, the number of fibres/mm^2^ counted from the optical images (92, Table 3) is probably more accurate than the number from the SEM images (75). In the micro-drop case, where objects are more homogeneously distributed, although the surface covered by optical images is 5 times larger (0.98 vs. 0.19 mm^2^) the number of counted single fibres is lower (1547 vs. 2275, Table 3) and this clearly reflects the higher resolution of electron microscopy over optical microscopy. In addition, as it is evident from the images given in Figures 4b, 8b and 9b, for highly homogeneous distribution of particles even a small area is representative of the whole sample.

**TABLE 3:** Manual counting of asbestos fibres in optical and SEM images collected on samples deposited with the drop and micro-drop methods, respectively.

Method	Drop		Microdrop	
	Microscopic	SEM	Microscopic	SEM
Number of studied images	52	26	14	2
Total inspected area (mm^2^)	3.59	2.40	0.98	0.19
Number of detected fibers	330	157	1526	364
fibers/mm^2^	92	65	1557	1916

**FIGURE 10:**
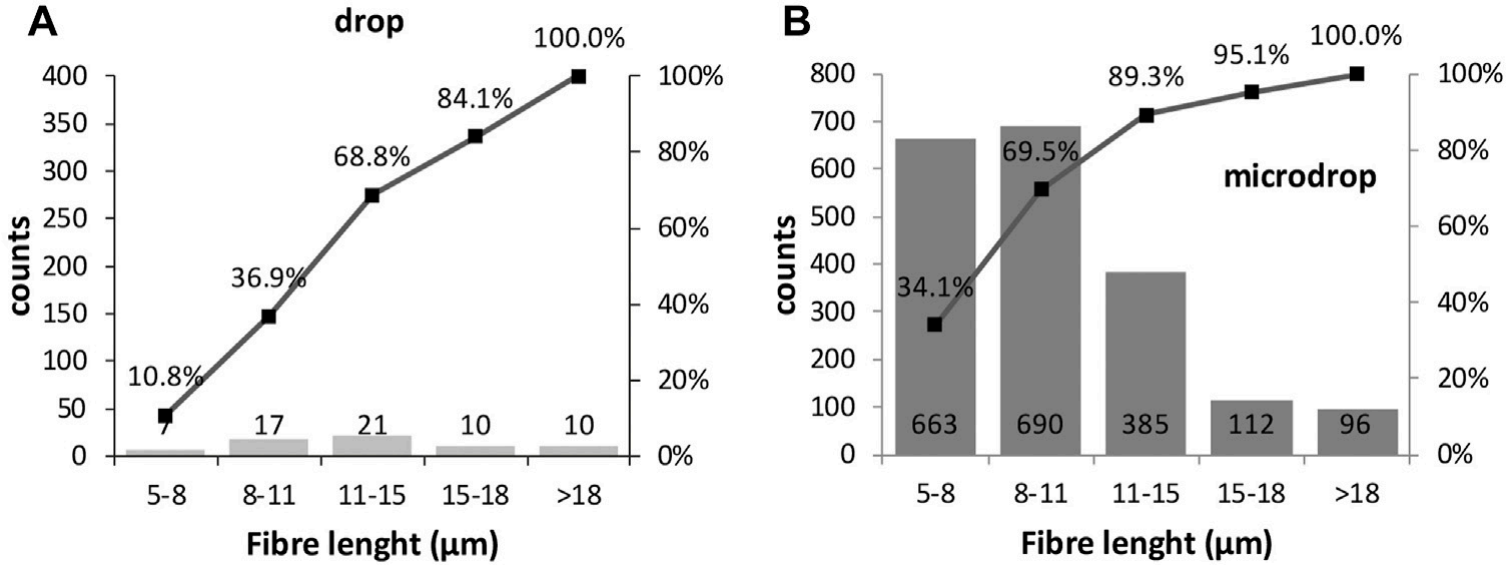
Histograms showing the length size distributions of the manually annotated fibres (see text for explanation) in **(A)** drop and **(B)** micro-drop depositions in SEM images. Counts are normalised to 1 mm^2^.

The distribution of the fibres lengths counted from SEM images (Figure 10) shows that the two classes 5–8 and 8–11 µm are almost equal and in total constitute 70% of the counted fibres (see cumulative trend over the histograms); their concentration gradually decreases towards longer dimensions for the micro-drop deposition. This result is in agreement with the strongly mono-dimensional nature of the used powder. For the drop samples very few particles with short length (5–11 µm classes) are counted, again stressing the superior reliability of the micro-drop deposition for *in vitro* tests.

## 4 Conclusion

Toxicological experiments are commonly carried out by depositing the material to be investigated on substrates where the cells are already present. A recent work (Yao et al., 2017) has demonstrated that for depositions made *via* micropipette there is an increasing impingement and formation of large bundles as a function of the increasing fibre amount in the suspension. These distributions have direct impact on the toxicity results, with a severe incertitude in the viability tests. We described here a more accurate deposition technique based on a micro-dispenser designed for inkjet printers and R&D applications and available on the market. This device allows depositing micro-sized droplets from a suspension of fibres dispersed in a liquid. With this approach both amount and spatial distribution of the fibres on the substrate can be efficiently controlled, being also highly reproducible. The statistical analysis of both optical and SEM images collected on depositions obtained using both methods starting from the same suspension showed that the distribution of particles is remarkably more homogeneous using the micro-drop technique and, in particular, the number of single fibres deposited *via* the micro-dispenser is far higher than that available by the micropipette technique.

## Data Availability

The original contributions presented in the study are included in the article/Supplementary Material, further inquiries can be directed to the corresponding author.
